# Supplementary material for: Multi‐dataset identification of innovative feature genes and molecular mechanisms in keratoconus
Source: J Cell Mol Med. 2024 Sep 19;28(18):e70079. doi: 10.1111/jcmm.70079 (PMC11412914; doi:10.1111/jcmm.70079)
Supplement: Supplementary file 2 — Material S2. [file JCMM-28-e70079-s002.zip › 7.GSEA analysis/4.MYRF/KEGG_PROSTATE_CANCER.html]

Details for gene set KEGG\_PROSTATE\_CANCER[GSEA]

|  || Dataset | MYRF.group.cls#h\_versus\_l |
| Phenotype | group.cls#h\_versus\_l |
| Upregulated in class | l |
| GeneSet | KEGG\_PROSTATE\_CANCER |
| Enrichment Score (ES) | -0.3744733 |
| Normalized Enrichment Score (NES) | -1.5882472 |
| Nominal p-value | 0.012024048 |
| FDR q-value | 0.25718212 |
| FWER p-Value | 0.632 |
Table: GSEA Results Summary

  

Fig 1: Enrichment plot: KEGG\_PROSTATE\_CANCER      
 Profile of the Running ES Score & Positions of GeneSet Members on the Rank Ordered List

  

| SYMBOL | TITLE | RANK IN GENE LIST | RANK METRIC SCORE | RUNNING ES | CORE ENRICHMENT || 1 | PIK3R5 | na | 661 | 0.528 | 0.0181 | No |
| 2 | INSRR | na | 1368 | 0.412 | 0.0268 | No |
| 3 | PDGFRA | na | 1428 | 0.407 | 0.0537 | No |
| 4 | AR | na | 1716 | 0.381 | 0.0723 | No |
| 5 | CCNE2 | na | 2609 | 0.317 | 0.0688 | No |
| 6 | KLK3 | na | 6644 | 0.174 | -0.0354 | No |
| 7 | SOS1 | na | 6706 | 0.172 | -0.0250 | No |
| 8 | PIK3R1 | na | 7105 | 0.161 | -0.0251 | No |
| 9 | IGF1R | na | 7135 | 0.160 | -0.0147 | No |
| 10 | SRD5A2 | na | 8005 | 0.138 | -0.0301 | No |
| 11 | BRAF | na | 8198 | 0.133 | -0.0262 | No |
| 12 | MTOR | na | 8431 | 0.129 | -0.0239 | No |
| 13 | IGF1 | na | 8743 | 0.121 | -0.0243 | No |
| 14 | KRAS | na | 9371 | 0.108 | -0.0348 | No |
| 15 | CREB3L3 | na | 9452 | 0.107 | -0.0296 | No |
| 16 | CREBBP | na | 9688 | 0.102 | -0.0292 | No |
| 17 | PIK3CG | na | 9794 | 0.100 | -0.0252 | No |
| 18 | AKT2 | na | 10210 | 0.092 | -0.0307 | No |
| 19 | CREB3L1 | na | 10715 | 0.083 | -0.0394 | No |
| 20 | PDPK1 | na | 11113 | 0.076 | -0.0455 | No |
| 21 | PTEN | na | 11228 | 0.075 | -0.0436 | No |
| 22 | CCNE1 | na | 11231 | 0.075 | -0.0384 | No |
| 23 | PIK3CA | na | 11255 | 0.074 | -0.0338 | No |
| 24 | E2F3 | na | 11256 | 0.074 | -0.0286 | No |
| 25 | HSP90AA1 | na | 12360 | 0.059 | -0.0563 | No |
| 26 | E2F2 | na | 12461 | 0.057 | -0.0552 | No |
| 27 | PIK3R2 | na | 12535 | 0.056 | -0.0534 | No |
| 28 | RB1 | na | 12759 | 0.053 | -0.0561 | No |
| 29 | SOS2 | na | 12783 | 0.053 | -0.0530 | No |
| 30 | TP53 | na | 12977 | 0.051 | -0.0550 | No |
| 31 | IKBKB | na | 12994 | 0.050 | -0.0520 | No |
| 32 | ERBB2 | na | 13228 | 0.047 | -0.0554 | No |
| 33 | MDM2 | na | 13578 | 0.043 | -0.0624 | No |
| 34 | PDGFA | na | 13993 | 0.038 | -0.0717 | No |
| 35 | MAP2K2 | na | 14012 | 0.038 | -0.0696 | No |
| 36 | CDKN1B | na | 14078 | 0.037 | -0.0688 | No |
| 37 | CHUK | na | 14348 | 0.034 | -0.0742 | No |
| 38 | MAPK3 | na | 14382 | 0.034 | -0.0728 | No |
| 39 | CCND1 | na | 14657 | 0.031 | -0.0785 | No |
| 40 | TCF7L2 | na | 15138 | 0.027 | -0.0905 | No |
| 41 | CTNNB1 | na | 15600 | 0.022 | -0.1023 | No |
| 42 | CREB3 | na | 15923 | 0.018 | -0.1103 | No |
| 43 | EGFR | na | 17031 | 0.008 | -0.1416 | No |
| 44 | PIK3CB | na | 17199 | 0.007 | -0.1460 | No |
| 45 | AKT1 | na | 18101 | -0.001 | -0.1719 | No |
| 46 | GSTP1 | na | 18399 | -0.004 | -0.1801 | No |
| 47 | CREB3L2 | na | 18517 | -0.005 | -0.1831 | No |
| 48 | EGF | na | 19142 | -0.012 | -0.2003 | No |
| 49 | GSK3B | na | 19466 | -0.016 | -0.2085 | No |
| 50 | MAPK1 | na | 20406 | -0.027 | -0.2338 | No |
| 51 | PIK3CD | na | 20433 | -0.027 | -0.2326 | No |
| 52 | PIK3R3 | na | 20464 | -0.027 | -0.2316 | No |
| 53 | HSP90AB1 | na | 20726 | -0.030 | -0.2370 | No |
| 54 | FGFR2 | na | 20983 | -0.033 | -0.2420 | No |
| 55 | TGFA | na | 21199 | -0.036 | -0.2457 | No |
| 56 | ATF4 | na | 21458 | -0.039 | -0.2504 | No |
| 57 | CREB3L4 | na | 21651 | -0.041 | -0.2531 | No |
| 58 | IKBKG | na | 21687 | -0.042 | -0.2511 | No |
| 59 | RAF1 | na | 21720 | -0.042 | -0.2491 | No |
| 60 | E2F1 | na | 21781 | -0.043 | -0.2478 | No |
| 61 | HRAS | na | 21856 | -0.044 | -0.2468 | No |
| 62 | CASP9 | na | 23052 | -0.060 | -0.2771 | No |
| 63 | FOXO1 | na | 23099 | -0.061 | -0.2741 | No |
| 64 | CREB1 | na | 23204 | -0.063 | -0.2727 | No |
| 65 | BAD | na | 23795 | -0.072 | -0.2846 | No |
| 66 | GRB2 | na | 24215 | -0.079 | -0.2911 | No |
| 67 | HSP90B1 | na | 24682 | -0.088 | -0.2984 | No |
| 68 | PDGFB | na | 26504 | -0.123 | -0.3423 | No |
| 69 | PDGFRB | na | 27235 | -0.141 | -0.3535 | No |
| 70 | EP300 | na | 27396 | -0.145 | -0.3479 | No |
| 71 | TCF7L1 | na | 27666 | -0.152 | -0.3450 | No |
| 72 | NFKB1 | na | 28241 | -0.168 | -0.3498 | No |
| 73 | ARAF | na | 28444 | -0.174 | -0.3434 | No |
| 74 | CDK2 | na | 29418 | -0.203 | -0.3572 | No |
| 75 | NRAS | na | 30019 | -0.224 | -0.3587 | Yes |
| 76 | TCF7 | na | 30107 | -0.227 | -0.3452 | Yes |
| 77 | MAP2K1 | na | 30150 | -0.229 | -0.3303 | Yes |
| 78 | INS | na | 30231 | -0.232 | -0.3163 | Yes |
| 79 | RELA | na | 31135 | -0.271 | -0.3233 | Yes |
| 80 | NFKBIA | na | 31525 | -0.292 | -0.3140 | Yes |
| 81 | PDGFD | na | 32544 | -0.354 | -0.3185 | Yes |
| 82 | BCL2 | na | 32817 | -0.374 | -0.3001 | Yes |
| 83 | LEF1 | na | 33408 | -0.432 | -0.2867 | Yes |
| 84 | CDKN1A | na | 33771 | -0.483 | -0.2632 | Yes |
| 85 | NKX3-1 | na | 34541 | -0.702 | -0.2360 | Yes |
| 86 | PDGFC | na | 34611 | -0.758 | -0.1846 | Yes |
| 87 | CREB5 | na | 34647 | -0.814 | -0.1283 | Yes |
| 88 | FGFR1 | na | 34693 | -0.924 | -0.0646 | Yes |
| 89 | AKT3 | na | 34696 | -0.932 | 0.0009 | Yes |
Table: GSEA details [plain text format]

  

Fig 2: KEGG\_PROSTATE\_CANCER      
 Blue-Pink O' Gram in the Space of the Analyzed GeneSet

  

Fig 3: KEGG\_PROSTATE\_CANCER: Random ES distribution      
 Gene set null distribution of ES for **KEGG\_PROSTATE\_CANCER**
